# How aminoglycosides are used in critically ill patients in a teaching hospital in North of Iran

**Published:** 2015

**Authors:** Ebrahim Salehifar, Gohar Eslami, Nematollah Ahangar, Mohammad Reza Rafati, Shafagh Eslami

**Affiliations:** 1 Department of Clinical Pharmacy, Faculty of Pharmacy, Thalassemia Research Center, Mazandaran University of Medical Sciences, Sari, Iran.; 2 Department of Clinical Pharmacy, Faculty of Pharmacy, Cardiovascular Research Center, Mazandaran University of Medical Sciences, Sari, Iran.; 3 Department of Pharmacology-Toxicology, Pharmaceutical Research Center, Faculty of Pharmacy, Mazandaran University of Medical Sciences, Sari, Iran.; 4 Faculty of Pharmacy, Mazandaran University of Medical Sciences, Sari, Iran.

**Keywords:** Rational use, Aminoglycosides, ICU, Antibiotics, DUE

## Abstract

**Background:**

Resistance to antimicrobial agents including aminoglycosides (AGs) is a great concern that is mainly related to inappropriate use. Since there were not adequate data regarding how rationally AGs are being prescribed in our critically ill patients, this study was conducted to determine the main issues in the area of appropriate use of this antibiotic class.

**Methods:**

One hundred patients who were in the intensive care units (ICUs) of Imam Khomeini Teaching Hospital from February 2012 to August 2012 were included. A data gathering form was prepared based upon the recommendations provided by Up-to-date (20.1) 2012 and Medscape 2013. All demographic characteristics and other information about time of beginning and duration of dosage, interval of administration of AGs and creatinine (Cr) level were collected. In statistical analysis, SPSS Version 16 software was used. Independent samples t-test was used to compare the quantitative and chi-square for qualitative variables.

**Results:**

Sixty six (66%) of patients received gentamicin and 38% received amikacin. In 27% of patients, serum creatinine (Cr) had been checked before and after AGs administration and 4 patients had no renal function monitoring. Monitoring of serum concentration and Cr clearance estimation was not carried out for any patients. Culture and laboratory sensitivity tests were done on 17 patients and E-coli (57%) was the most common isolated organism.

**Conclusion:**

The results of this study revealed that majority of the hospitalized patients in the ICU and the dosage of AGs had not been adjusted to renal function.

**A**ntibiotic resistance is a major global problem particularly in developing countries (1, 2). Inappropriate and irrational prescription of antimicrobial agents causes an increase in microbial resistance. Based on the definition of World Health Organization (WHO), the rational use of medicines means prescribed medications with the effective dose and the correct indication in time, to create the lowest possible cost to the patient and society (3, 4). Aminoglycosides (AGs) are antimicrobial drugs, which have been used since the 1940’s and as a result of a widespread consumption, an increasing resistance to them was observed (1). AGs are among the potent broad spectrum drugs used in the treatment of life-threatening infections specifically gram-negative bacilli infections, and with the inhibition synthesis of S30 ribosome and the inhibition of bacteria protein synthesis cause a disturbance in the monotony of the cell wall of the bacteria and ultimately leads to bacterial cell death (5, 6). 

Several factors must be assessed for the reasonable consumption of these medicines which include: indication, dosage, medication level measurements, and the duration of treatment (7, 8).

 Because of the narrow therapeutic index and the high chance of side effects in AGs, these factors are of great importance for this group of antimicrobial agents (8, 9). In spite of great importance of the use of AGs in critically ill patients, the pattern of AGs administration in our setting has not been investigated yet. The aim of this study was to determine how AGs are used in our setting. 

## Methods

This study was conducted in an intensive care unit (ICU) of Imam Khomeini Hospital, a referral teaching center affiliated to Mazandaran University of Medical Sciences, North of Iran. This study was approved by a research committee under the supervision of Research and Technology Deputy vice- Chancellor of the university. 

The patients hospitalized during February 2012 to August 2012 and received AGs in their current hospitalization were entered in the study. A data gathering form was prepared based on the recommendations provided by Up-to-date (20.1) 2012 and Medscape 2013 to extract relevant demographic and clinical characteristics of patients.

 The beginning date and dosage of AGs administration, frequency of dosage, and duration of treatment corresponded with drug monitoring (TDM). 

**Statistical analysis**

SPSS software Version 16 was used for statistical analysis. The quantitative variables were evaluated using the independent sample t-test and the qualitative variables using the chi-square test. A p-value less than 0.05 was reported as statistically significant.

## Results

In this study, a total of one hundred patients (32 females and 68 males) were enrolled in the study. The patient’s demographic and clinical data are presented in table 1. The age in both sexes and also the average length of hospital stay in days for patients were not different. The most frequent cause of hospitalization was traffic accidents which account for 42% and the most commonly used antibiotics were cephalosporins. 

**Table 1:** Demographic and clinical characteristics of patients

**n (%)**	**Variables**
**Sex**	
Male Female	68 (68%) 32 (32%)
**Age (mean±SD)**	
Male Female	51.7±22.5 45.5±16.5
	P=0.16
**Hospital stay in days (mean±SD)**	
Male Female	16.7±12.4 13.9±12
	P=0.29
**Cause of hospitalization**	
Accidents BPH Neurosurgery Gynecology Neoplasm Falling Others	42 (42%) 14 (14%) 11 (11%) 17 (17%) 6 (6%) 6 (6%) 4 (4%)
**Medical history**	
No previous history Hypertension Diabetes Mellitus Hypertension and Diabetes Mellitus Depression Others	64 (64%) 12(12%) 6 (6%) 9(9%) 2 (2%) 6 (6%)
**Clinical Sign/Symptoms**	
Asymptomatic Leukocytosis Fever Sputum Dyspnea Dysuria Fever and Leukocytosis Fever and Dysuria Other	44 (44%) 12 (12%) 10 (10%) 9 (9%) 7 (7%) 5 (5%) 5 (5%) 4 (4%) 4 (4%)
**Received antibiotics**	
Gentamicin Cefazolin Amikacin Vancomycin Ceftriaxone Imipenem Clindamycin Injected Ciprofloxacin Cephalexin	66 (66%) 69 (69%) 38 (38%) 37 (37%) 32 (32%) 24 (24%) 18 (18%) 14 (14%) 14 (14%)

Forty-four percent of patients had no signs of infection at the time of starting antibiotics and leucocytosis (12%) was the most prevalent sign, followed by fever and sputum. Cefazolin, vancomycin, ceftriaxone and imipenem were the most common antibiotics administered with AGs, which occurred in 69%, 37%, 32% and 24% of cases, respectively (table 1). Average treatment duration and start time of antibiotics have been demonstrated in table 2. Antibiotics were administered in most of hospital stay days (13.45 days versus15.8 stay days) and AGs were administered in almost half of days when patients were on antibiotics (7 days versus 13.45 days) (table 2). 

**Table 2:** Start time and average treatment duration with antibiotics

**Variables**	**Mean** **±** **SD (days)**
Start time of antibiotics	2.96±2.85
Duration of treatment with antibiotic	13.45±12.38
Duration of hospital stay	15.8±12.27
Start time of aminoglycosides	5.2±5.47
Duration of treatment with aminoglycosides	7±6.1

Table 3 shows the comparison of serum creatinine (Cr) in both genders, before and after receiving aminoglycosides. Serum Cr was measured for only 27 patients both before and after receiving AGs. In male patients, Cr changes were statistically different (P= 0.03) (table 3). 

**Table 3:** Mean serum creatinine before and after receiving aminoglycosides

	**Before AG** **(mean±SD)**	**After AG** **(mean±SD)**	**95% Confidence Interval **	**P-value**
Female (n=5)	0.820±0.21	0.70±0.07	-0.22 to 0.46	0.39
Male (n=22)	1.02±0.317	1.15±0.462	-0.25 to 0.01	0.03
Total (n=27)	0.985±0.308	1.07±0.45	-0.19 to 0.02	0.13

AG: aminoglycoside

In four patients, no evaluation of kidney function test was carried out during their ICU stay. In addition, in 37 patients before receiving AGs and in 52 patients after receiving AGs, no kidney function tests were done. In 28 patients, renal clearance was less than 60 mL/min, and this was observed in 7 of them before starting AGs. In addition, no dose adjustments were done in 18 (64.3%) of them.

Culture and sensitivity tests were carried out on 17 (17%) of patients including 8 urine, 8 sputum and 1 blood culture. Seven (41%) samples were associated with bacterial growth. In 4 (57%) of positive samples, E-coli was grown (1 urine and 3 blood cultures). Entrobacteria was isolated in the remaining 3 patients which was sensitive to cotrimoxazole, cefixime, gentamicin, nitrofurantoin, ceftazidime and nalidixic acid.

## Discussion

The aim of this study was to examine the use of AGs at the ICU of Imam Khomeini teaching Hospital. This study shows that a significant percentage of patients (44%) were prescribed antibiotics even though they had no sign of infections. Despite the importance of kidney monitoring in prescribing AGs, creatinine clearance calculation and further dose adjustment were not accomplished for any patient (at least, there was not any documented evidence in the medical record). Moreover, in 4% of patients, routine kidney monitoring (BUN and creatinine measurement) was not done.

Various studies demonstrated the inappropriate antibiotic prescribing in half of the patients admitted to the hospital (7, 8, 10). AGs are broad spectrum antibiotics with narrow therapeutic index. In the treatment of life-threatening infections such as endocarditis, fever with neutropenia and also infections with gram negative bacillus (*Pseudomonas aeruginosa*), AGs are recommended for 7-10 days. Indeed, it has been mentioned that after 10 days, the side effects will be more than the benefits (3, 11). In our study, 18% of patients received AGs for more than 10 days, which is similar to the Arshadi’s study (3). 

Considering that our study was carried out in intensive care units, a longer treatment period is not far from our expectations. However, due to the increased risk of nephrotoxicity and ototoxicity, in particular after 10 days of treatment with these medications, additional monitoring including therapeutic drug monitoring, precise evaluation of renal function and if possible, hearing function is important (1, 12). A 25% increase in creatinine was reported previously by Zahar et al. (8). In our study, a statistically significant difference occurred for the male patients, though this was not significant when data pooled for both sexes. This may be explained by this fact that creatinine measurement was done on only a few patients. 

In this study, kidney function evaluation was not carried out on 4% of patients and creatinine clearance was not measured for any of the patients. Due to the importance of measuring patient’s creatinine clearance at the beginning and then every 1 to 3 days after starting AGs (13, 14), regular renal function monitoring and creatinine clearance measurements based on the patient’s weight are recommended. The bactericidal effects of antibiotics are related to concentration and post antibiotic effects (PEAs), as a result, a single dose prescription provides enhanced effectiveness, lower resistance and fewer side effects (1). AGs are antibiotics with bactericidal properties dependent on either concentration or postantibiotic effect (PAE) and in consequence many studies recommend a single daily dose, wherein adequate effectiveness and lower complications are expected (14, 15). 

In several studies, a single daily dose regimen in the intensive care unit was evaluated, which was accompanied with better effectiveness and fewer side effects in particular renal toxicity (16-20). In current study, the single daily dose method was not administered to any of the patients. Regarding the consumption of other nephrotoxic drugs with AGs in critically ill patients, single daily dose administration is more justified. In patients with critical conditions, a higher dose of AGs is needed to achieve the normal therapeutic level due to the different kinetic parameters (increasing volume of distribution, reducing the clearance and albumin). In several studies, different pharmacokinetic behaviors of AGs in critically ill patients have been noted. Increased volume of distribution, alteration of albumin level and different clearances especially in trauma patients are among the different kinetic characteristics of AGs. In most studies, measuring the level of AGs has been advised and dose should be individualized according to patient-specific level (20, 21). 

In the case of a treatment lasting more than 10 days, old age, concomitant nephrotoxic medications and underlying diseases, blood level measurements of AGs are warranted (14). Considering these criteria, checking the blood level in 28% of the patients in our study was essential, however, the blood levels of the medications were not checked in any of the cases. The high cost of measuring the blood levels especially in the private section and also the lack of expertise to operate most laboratory facilities, and teaching hospitals are seen responsible for this.

Preparing guidelines for the rational administration of antibiotics, particularly in the ICU, is one approach for a more rational use of antimicrobial agents including AGs. In a recent published study, the preparation of these guidelines has been carried out with the cooperation among intensive care specialists, infectious disease specialists and pharmacists. Cooperation between various specialists, in particular, the use of clinical pharmaceutical consultants in special care units can play an important role in appropriate effectiveness, reduced toxicity, in reducing resistance and a more rational consumption of these medicines (21, 22).

Due to the significant role of the irrational consumption of AGs on antibiotic resistance, initiation or continuation of treatment based on culture results is essential. In this study, we found that antibiotics were prescribed to all patients empirically. The rate of empiric therapy in other studies was variable including 43% and 94%, as reported by Arshed et al. and Ceyhan et al., respectively (3, 13). The initiation of appropriate antibiotics in accordance to antibiotic resistance and patients’ clinical situations are inevitable, especially in ICUs. In this study and also in another study in children in Mazandaran province (23), E. coli was the most common microorganism isolated from sample cultures and in spite of resistance to gentamicin (50%), this was administered. In addition, in one patient (25%) resistance to amikacin was observed. 

In conclusion, the most important defects observed in this study regarding the use of AGs were high rate of empiric therapy without culture and sensitivity test, no calculation of creatinine clearance and the inappropriate dose adjustment in a large number of patients. Discussing the results of this study in the Drug and Therapeutic Committee of the hospital and also using standard guidelines for the rational use of AGs can be effective in improving the situation. 

## Notes

**Funding:** This study was supported by a grant from the Research and Technology Deputy Vice-Chancellery of Mazandaran University of Medical Sciences.
